# Tough Hydrogels with Different Toughening Mechanisms and Applications

**DOI:** 10.3390/ijms25052675

**Published:** 2024-02-26

**Authors:** Zhengyu Xu, Yanru Chen, Yi Cao, Bin Xue

**Affiliations:** 1 Collaborative Innovation Center of Advanced Microstructures, National Laboratory of Solid State Microstructure, Department of Physics, Nanjing University, Nanjing 210093, China; dz1922031@smail.nju.edu.cn (Z.X.); mg21220197@smail.nju.edu.cn (Y.C.); 2 Jinan Microecological Biomedicine Shandong Laboratory, Jinan 250000, China

**Keywords:** hydrogels, high toughness, sacrificial bonds, hierarchical architecture, network topology, force-triggered length release, tissue engineering, flexible electronics

## Abstract

Load-bearing biological tissues, such as cartilage and muscles, exhibit several crucial properties, including high elasticity, strength, and recoverability. These characteristics enable these tissues to endure significant mechanical stresses and swiftly recover after deformation, contributing to their exceptional durability and functionality. In contrast, while hydrogels are highly biocompatible and hold promise as synthetic biomaterials, their inherent network structure often limits their ability to simultaneously possess a diverse range of superior mechanical properties. As a result, the applications of hydrogels are significantly constrained. This article delves into the design mechanisms and mechanical properties of various tough hydrogels and investigates their applications in tissue engineering, flexible electronics, and other fields. The objective is to provide insights into the fabrication and application of hydrogels with combined high strength, stretchability, toughness, and fast recovery as well as their future development directions and challenges.

## 1. Introduction

A hydrogel is a type of polymer network with a three-dimensional structure that contains hydrophilic groups and can swell in water, typically with a water content ranging from 50% to 99.9%. Hydrogels are characterized by good biocompatibility, the ability to regulate crosslinked networks, responsiveness to external stimuli, and molecular permeability [1]. They find wide-ranging applications in tissue engineering [2,3,4], adhesive materials [5,6], drug delivery [7,8], artificial muscles [9], different types of sensors [10,11,12], soft robots [13,14], brain-computer interfaces [15], and other fields. Currently, most traditional hydrogels are soft and brittle, with mechanical properties such as fracture stress, fracture strain, and fracture toughness far lower than natural materials. This low toughness is attributed to the uneven topology of their crosslinked networks, which lack effective energy dissipation mechanisms to prevent crack propagation and stress concentration. Consequently, when subjected to external forces, the stress in the hydrogel quickly concentrates on structural defects or weak crosslinking points, leading to rapid crack expansion and eventual failure, severely limiting their practical applications [16,17,18]. Additionally, there is often a trade-off between strength and toughness in hydrogels, as these properties are mutually exclusive [19].

Designing hydrogels with high toughness is key to addressing the challenges and has become a hot research topic. Tough hydrogels exhibit excellent ductility, high toughness, pressure resistance, and self-healing ability [20,21]. The density and dynamic characteristics of crosslinking points [22,23], the uniformity of the network structure [24], and the density and flexibility of the polymer chain [25,26], among other factors, all affect the mechanical strength of hydrogels. In addition, the special design of the hydrogel network structure is also crucial for improving the mechanical properties of hydrogels. Common methods to enhance the toughness of hydrogels include introducing sacrificial units to dissipate energy, such as double-network hydrogels and some single-network hydrogels [27,28,29,30]; increasing the crosslink density by enhancing the functionality of single crosslink points, as seen in nanocomposite hydrogels [31,32]; improving the uniformity of the hydrogel crosslinked network [33]; and introducing “molecular sliding mechanisms” or strong dynamic interaction systems, such as slide-ring hydrogels and some single-network hydrogels [34,35]. These different design strategies can also be combined to fabricate composite hydrogels with excellent mechanical properties, which have been studied and discussed in depth by researchers before [36,37,38]. Compared with previous reviews, this review comprehensively discusses the design principles and toughening mechanisms of tough hydrogels, as well as classifies them according to their characteristics. This review also introduces the mechanical properties of various tough hydrogels and their applications in tissue engineering, 3D printing, flexible electronic devices, and so on. Furthermore, we also discussed the remaining challenges of tough hydrogels and look forward to future development directions and challenges.

## 2. Strategies and Properties of Tough Hydrogels

### 2.1. Toughening Hydrogel with Sacrificial Bonds

**Figure 1:**
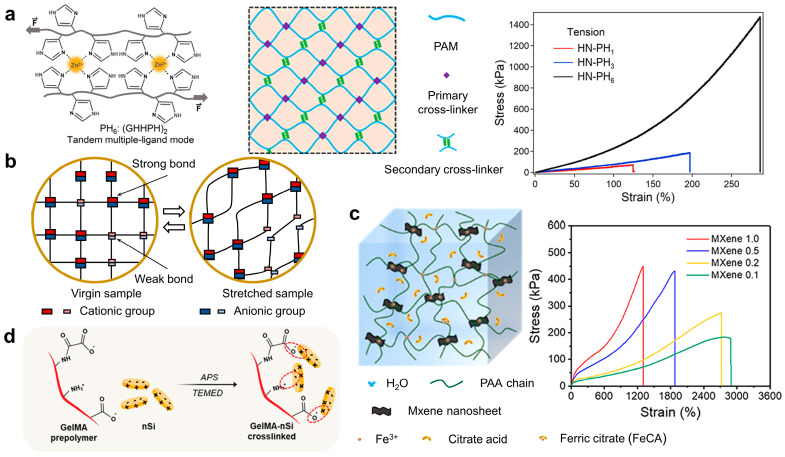
Schematic of single-network hydrogels toughening with sacrificial bonds. (**a**) Molecular engineering of metal coordination interactions for strong, tough, and fast-recovery hydrogels. Reprinted with permission from Ref. [45]. 2020, American Association for the Advancement of Science. Schematic illustration of metal ion coordination complexes formed by two metal chelation sites in tandem (PH_6_); schematic illustration of the network structure of HN-PH_6_ hydrogels and uniaxial stress-strain curves under tension. (**b**) Schematic of the structure of physically crosslinked hydrogel composed of polyampholytes. During the deformation of hydrogels, the strong ionic bonds act as permanent crosslinkers, similar to covalent bonds, while weak ionic bonds serve as reversible sacrificial crosslinkers, adapted from Ref. [46]. (**c**) Schematic of the highly stretchable and tough nanocomposite physical hydrogels, which contain MXene nanosheets that served as multifunctional crosslinkers and effective stress transfer centers, as well as the stress–strain curves. Reprinted with permission from Ref. [55]. 2022, American Chemical Society. (**d**) The components involved in the chemical crosslinking of the mechanically tough nanocomposite hydrogel. The electrostatic interaction between GelMA and nSi is highlighted. Reprinted with permission from Ref. [56]. 2023, American Chemical Society.

The most commonly used method to enhance the toughness of hydrogels is through sacrificial bonds, which include single-network hydrogels and double-network hydrogels. Single-network hydrogels have a simple structure and are earlier developed hydrogel network systems with limited tensile properties and toughness [39,40]. To improve their tensile properties and toughness, a non-covalent crosslinking network structure can be introduced, resulting in a hydrogel with two different crosslinking mechanisms [41,42,43,44]. The covalent crosslinking points provide stability to the mechanical structure and matrix modulus, while the reversible secondary crosslinking points on the polymer chain segments or side chains act as the main energy dissipation mechanism to enhance hydrogel toughness. For instance, Cao et al. developed strong, tough, and fast-recovery hydrogels by incorporating a specially designed peptide-metal complex as the physical crosslinker, as shown in Figure 1a. The decapeptide used in their study contained two tandem zinc-binding motifs and had high thermodynamic stability, strong binding strength, and a rapid binding rate with Zn^2+^. This peptide-metal complex enabled fast binding and unbinding kinetics. Moreover, the presence of multiple ligands within each binding site and the cooperative arrangement of binding sites enhanced the stability and strength of the hydrogels. As a result, the engineered hybrid network hydrogels, containing the peptide-zinc complex, exhibited a break stress of approximately 3.0 MPa, a toughness of about 4.0 MJ m^−3^, and fast recovery within seconds [45]. Additionally, Gong et al. introduced a hydrogel with ionic bonds of varying strengths, formed through the electrostatic interaction between two oppositely charged polyelectrolytes (3-(methacrylamido)propyl-trimethylammonium chloride (MPTC) and sodium p-styrenesulfonate (NaSS)). As shown in Figure 1b, the strong bonds act as permanent crosslinking points, providing sufficient matrix elasticity, while the weak bonds reversibly dissociate and dissipate energy. This hydrogel exhibits a fracture energy of 4 kJ m^−2^ [46]. Furthermore, Sheiko et al. designed a N,N-dimethylacrylamide and methacrylic acid couple (DMAA-co-MAAc) copolymer hydrogel utilizing the collaborative crosslinking of hydrogen bonds and covalent bonds. It exhibits a Young’s modulus of up to 28 MPa and a toughness of up to 93 kJ m^−2^. This hydrogel also shows fatigue resistance and restorability, demonstrating the feasibility and advantages of combining reversible hydrogen bonding and covalent bonding in preparing stretchable and tough hydrogels [47]. Single-network hydrogels with high toughness can also be created solely through physical interactions, including ionic interactions, hydrogen bonds, hydrophobic interactions, metal coordination interactions, protein-specific interactions, and so on [48,49,50,51,52]. Strong physical interactions ensure the physical properties of hydrogels, while reversible dynamic physical interactions provide self-healing properties to hydrogels [53]. For instance, Sherman et al. utilized the host–guest interaction between acrylamide and cucurbituril as a reversible sacrificial bond to dissipate energy and prepared a supramolecular hydrogel with a tensile strain of 107 times. This hydrogel also exhibits significant self-healing properties and conductivity at room temperature [54].

Nanocomposite hydrogels are systems formed by using nanomaterials as high-functionality crosslinking points to connect multiple polymer chains [57,58]. Nanomaterials encompass inorganic salt particles, carbon-based nanomaterials, metal nanomaterials, polymer colloids, and so on [59,60]. The surface of nanomaterials used as crosslinking points in nanocomposite hydrogels is easier to be modified and connected with a variety of polymer chains, compared with that of traditional crosslinking networks. When stretched, a small amount of polymer chains in these hydrogels will break and consume energy without affecting the elongation of the main chain. As a result, nanocomposite hydrogels exhibit excellent tensile properties and high toughness. For example, Li et al. designed a polyacrylamide/calcium hydroxide (Ca(OH)_2_) nano-spherulites (PAM/CNS) nanocomposite gel with extremely strong stretchability and ultra-high toughness based on the polyacrylamide and low-concentration, small-sized calcium hydroxide nanospherulites [61]. Some nanocomposite hydrogels use nanowires or nanotubes as crosslinking points, such as carbon nanotubes, assembled nanowires, and so on [62,63]. These hydrogels not only exhibit excellent stretchability and high toughness but also possess photoelectric or other special properties. They can be used in applications such as drivers, conductive tapes, biosensors, artificial skin, and other fields. The solubility or stability of carbon-based materials can be enhanced by modifying polar groups or stabilizers on the surface, including amino groups, carboxyl groups, proteins, pyrene molecules, and so on. Subsequently, the modified carbon-based materials can be connected with polymer chains [64]. For example, Liu et al. synthesized a nanocomposite hydrogel based on the hydrogen bonding of nanotubes and poly-acryloyl-6-amino caproic acid, which has excellent stretchability and self-healing properties [65]. In addition, Ma et al. constructed injectable antibacterial conductive cryogels based on carbon nanotube (CNT) and glycidyl methacrylate functionalized quaternized chitosan. These gels exhibit robust mechanical strength, rapid blood-triggered shape recovery, and absorption speed, as well as high blood uptake capacity, indicating their great potential for use in noncompressible hemorrhage hemostasis dressing and wound healing applications [66]. Nanosheets or nanoribbons are utilized as crosslinking points in two-dimensional nanocomposite hydrogels, including clay nanosheets, graphene sheets, and so on [5]. In 2002, Haraguchi et al. peeled the synthesized clay into nanosheets and then modified hydrophilic monomers on the surface. Subsequently, they prepared novel nanocomposite hydrogels with a unique organic–inorganic clay network structure through a free radical polymerization [67]. They further studied the properties of nanocomposite hydrogels using clay nanosheets as crosslinking points and optimized the tensile properties and toughness by preparing polymer brushes or adsorbing polymer chains on the surface of the clay sheets. Additionally, Lu et al. reduced graphene oxide into conductive graphene using polydopamine and then formed PDA-pGO-PAM hydrogels based on the covalent and physical interactions of polyacrylamide and conductive graphene. This hydrogel exhibits extremely high tensile properties, excellent electrical conductivity, ductility, high toughness, viscosity, self-healing properties, biocompatibility, and more. Consequently, it can be used in implantable bioelectronic devices and other fields [68]. In another study, as shown in Figure 1c, Xie et al. proposed highly stretchable high-toughness nanocomposite physical hydrogels based on a multibond network design rationale, which contain 2D Ti_3_C_2_T_x_ MXene nanosheets served as multifunctional crosslinkers and effective stress transfer centers. These conductive MXene and numerous ions enable hydrogels to have high toughness, superior conductivity, strain sensitivity, and self-healing performance, suggesting great potential for use in intelligent flexible electronics [55]. As depicted in Figure 1d, Arghya et al. constructed a mechanically tough nanocomposite hydrogel for pharmaceutical drug delivery by exploiting the electrostatic interaction between nanosilicates (nSi) and gelatin methacrylate (GelMA). Notably, this hydrogel is prepared at subzero temperatures and possesses macroporous structures, rendering it suitable for cell infiltration [56]. In addition, vitrimers containing reversible covalent bonds can also enhance the mechanical properties of hydrogels [69,70]. For example, Cedric developed a type of vitrimer gel based on transesterification through the epoxy ring-opening reaction with carboxylic acids of functionalized poly (ethylene glycol) (PEG) and triglycidyl ether crosslinkers. It exhibits a good maximum strain (300%), high conductivity, and full recovery, allowing it to be used as a strain sensor, soft electrode, and ionic cable [71].

**Figure 2:**
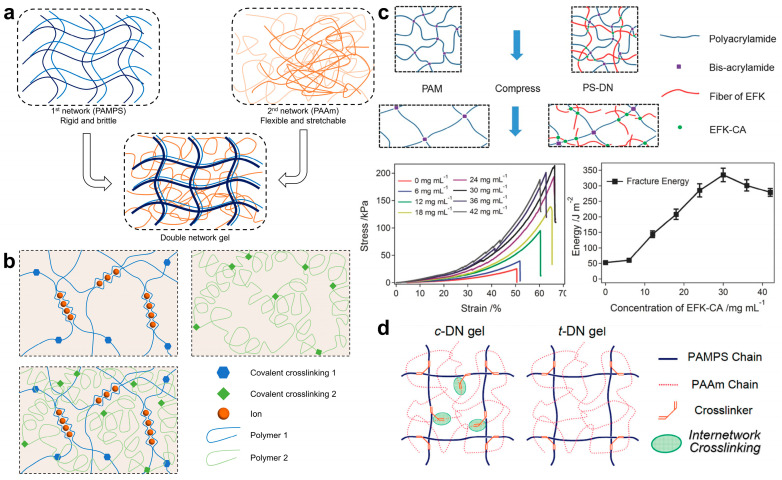
Schematic of double-network hydrogels toughening with sacrificial bonds. (**a**) PAMPS-PAAm covalent double-network hydrogels, adapted from Ref. [75]. (**b**) An alginate-polyacrylamide hybrid gel based on the ionic crosslinking of alginate and the covalent crosslinking of acrylamide, adapted from Ref. [21]. (**c**) A polymer-supramolecular polymer double-network hydrogel based on the combination of a polyacrylamide covalently crosslinked network and a self-assembled polypeptide fiber supramolecular network EFK, as well as the compression-fracture curves and fracture energies at different EFK-CA (EFK peptide with a cinnamic acid) concentrations. Reprinted with permission from Ref. [77]. 2016, John Wiley and Sons. (**d**) Truly independent double-network gels and double-network gels with two networks interconnected. Reprinted with permission from Ref. [78]. 2009, American Chemical Society.

A double-network hydrogel consists of two interpenetrating polymer networks that can be chemically or physically crosslinked and are independent of each other. This method is widely used to prepare highly tough hydrogels by designing a unique energy dissipation mechanism in the crosslinking network of double-network hydrogels [72,73,74]. A fully chemically crosslinked double-network hydrogel, also known as a covalent double-network hydrogel, consists of two polymer networks connected by chemical bonds that are independent of each other. Osada et al. first proposed a covalent double-network hydrogel through two-step continuous free radical polymerization in 2003, in which the primary network is a dense rigid network and the secondary network is a loose flexible network. The rigid polyelectrolyte network poly(2-acrylamido-2-methylpropanesulfonic acid) (PAMPS) was first polymerized and then the primary network was immersed in an aqueous solution containing neutral monomers. Then, the loose secondary high molecular weight polyacrylamide covalent network PAAm was polymerized in the swollen primary network. Finally, a PAMPS-PAAm double-network hydrogel containing intertwined loose long chains and dense short chains was obtained, as shown in Figure 2a [75]. The primary polyelectrolyte network undergoes extreme swelling, causing it to break into small cluster structures that serve as sliding crosslinking points in the secondary loose network. The rupture of covalent bonds in the short chains consumes a significant amount of energy, providing the hydrogel with mechanical strength. Meanwhile, the loose secondary network structure gradually straightens from its curled and entangled state, contributing to the ductility of the hydrogel. The synergistic effect of these two different covalent networks enables the hydrogel to dissipate energy under large strains, resulting in high toughness and excellent tensile properties. These properties are much better than those of traditional single polyelectrolyte hydrogels or polyacrylamide hydrogels [73]. Lu et al. fabricated an NPs-pectin-polyacrylic acid adhesive hydrogel using Ag-Lignin nanoparticles (NPs) to initiate dynamic redox catechol chemistry. The presence of silver lignin nanoparticles establishes a sustained reductive-oxidative environment within the hydrogel network. Continuously generated catechol groups enable the hydrogel to preserve its adhesive properties, while also demonstrating exceptional resistance to stretching and high toughness. Additionally, this hydrogel exhibits favorable cell compatibility and shows high antibacterial activity, thereby holding potential for application in tissue engineering [76].

Covalent double-network hydrogels typically dissipate energy through the irreversible breakage of the first-level network. However, this toughening mechanism renders them unable to recover from initial damage and lacking in anti-fatigue properties, thus impacting their potential applications in biological scaffolds, cartilage repair, and so on. To address these limitations, researchers have focused on introducing dynamic physical crosslinked networks to replace the first-level “sacrificial network” in covalent double-network hydrogels. These dynamic physical networks utilize various mechanisms such as ionic pairing [21,49,79], hydrophobic interactions [80], hydrogen bonding [81], coordination interactions [82,83,84], and host–guest interactions. These double-network hydrogels are characterized by their high toughness, exceptional stretch resistance, fatigue resistance, self-healing ability, and other properties [85]. For example, Suo et al. proposed an alginate-polyacrylamide hybrid gel based on the ionic crosslinking of alginate and the covalent crosslinking of acrylamide, as shown in Figure 2b. These two crosslinked networks are covalently linked through amino groups and carboxyl groups [21]. The ionic crosslinked network is capable of dissipating energy through reversible dissociation, while the covalent network of the hydrogel inhibits crack expansion, thereby ensuring the high toughness of the hydrogel. This study provides a model for exploring the mechanisms of hydrogel deformation and energy dissipation. Additionally, inspired by the unique polymer-supramolecular polymer double-network (PS-DN) architecture found in natural articular cartilage [86], Wang et al. developed a PS-DN hydrogel based on the combination of a polyacrylamide covalently crosslinked network and a self-assembled polypeptide fiber supramolecular network EFK. This hydrogel dissipates energy by employing the EFK polypeptide fiber network as a “sacrificial” bond during compression and has high toughness, as illustrated in Figure 2c. Upon release of the force load, the polypeptides rapidly reassemble, endowing the hydrogel with rapid self-repair capabilities. This makes it a potential candidate for high-toughness materials in the regeneration of load-bearing tissues such as cartilage [77]. In most cases, a physical crosslinked network is dynamic and easy to break under strains, and the overall mechanical properties of physical crosslinked double-network hydrogels are still limited. However, Cao et al. have reported that tuning the network structures can improve the mechanical properties of these hydrogels, potentially offering new insights into combining high mechanical strength and fast recovery in physically crosslinked double-network hydrogels [87]. In addition, Gong et al. synthesized a series of physically crosslinked double-network hydrogels based on amphiphilic triblock copolymers, which contain strong hydrophobic domains and sacrificial dynamic bonds of hydrogen bonds. These hydrogels are stiff, and tough, and feature improved self-healing and self-recovery abilities [88]. Another approach to constructing hybrid double-network hydrogels involves introducing microcrystalline structures into covalent single-network hydrogels [89,90]. For example, Suo et al. proposed a crystallized polyvinyl alcohol/polyacrylamide (PVA-PAAm) gel utilizing a dry annealing method, which can dissipate energy through the reversible disassembly of the high-density polyvinyl alcohol microcrystalline physical network. As a result, this hydrogel possesses high mechanical strength and toughness. In addition, the polyacrylamide covalent network provides matrix elasticity for this hydrogel [90]. Another research focus is on developing biomimetic double-network hydrogels based on the characteristics of biopolymers in nature. For example, Chen et al. synthesized highly mechanical and recoverable double-network hydrogels through a one-pot synthesis approach. The agar molecules can form a double helix structure at room temperature and assemble into a thick helical bundle physical crosslinked network due to heat sensitivity. They will break into small cluster structures to dissipate energy when the hydrogels are stretched. Then, the agar molecules can return to their original state at high temperatures and enable the hydrogel to possess self-healing properties. The fracture strain can reach 20 times the original length [91]. As shown in Figure 2d, Gong et al. have also contributed to the field by demonstrating the anomalous high strength some double-network gels can attain when the second network is polymerized without any crosslinkers. This is due to the interconnection between the two networks through covalent bonds, and they named these kinds of double-network gels c-DN gels. They also synthesized truly independent double-network gels, which do not have covalent bonds between the first and the second networks, and named them t-DN gels. They demonstrated that t-DN gels cannot be toughened by the uncrosslinked second network [78].

### 2.2. Toughening Hydrogel with Hierarchical Architecture

**Figure 3:**
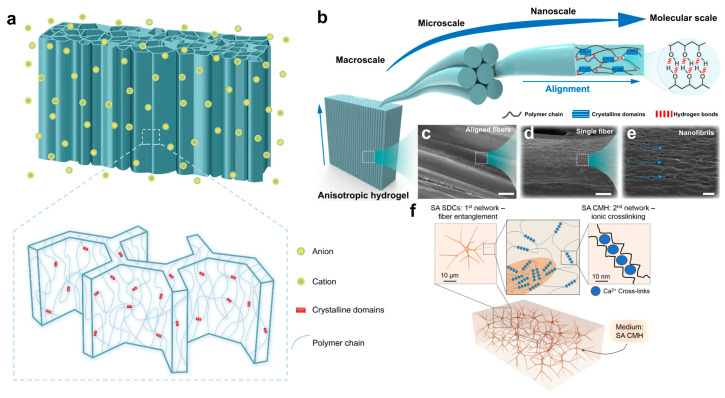
Schematic of high-toughness hydrogels toughening with hierarchical architecture. (**a**) A multi-length-scale hierarchical hydrogel prepared through freezing-assisted salting-out treatment, adapted from Ref. [102]. (**b**–**e**) Schematic illustration of anisotropic hydrogels with multiscale hierarchical architectures from macroscale to molecular scale and multiscale magnified SEM images showing the preferentially aligned fiber structures. The scale bars for (**c**–**e**) are 100, 10, and 2 μm, respectively. Reprinted with permission from Ref. [105]. 2024, Springer Nature. (**f**) Schematic of the hierarchical interactions between the colloidal and molecular alginate networks in a class of self-reinforced homocomposite hydrogels. Reprinted with permission from Ref. [106]. 2021, Springer Nature.

Natural structural materials often possess excellent mechanical properties resulting from their complex hierarchical assembly across multiple length scales. Therefore, it is possible and challenging to develop hydrogels with multi-level special network architectures, which can significantly enhance their mechanical properties for various applications, such as bioengineering [92,93,94,95,96,97,98,99,100,101]. He et al. introduced a strategy to create a multi-length-scale hierarchical hydrogel architecture utilizing a freezing-assisted salting-out treatment. The produced poly(vinyl alcohol) hydrogels exhibit excellent ultimate stress, high strain levels, and high toughness, benefiting from the highly anisotropic structures. These structures consist of micrometer-scale honeycomb-like pore walls, which are composed of interconnected nanofibril meshes, as depicted in Figure 3a. The properties of these hydrogels are even superior to those of natural tendons with the same water content. These strategies can also be applied to other polymers [102]. For example, He et al. also constructed a stretchable conducting tough hydrogel through an ice-templated, low-temperature polymerization (ITLP) strategy. This hydrogel has a hierarchical dendritic microstructure with mitigated nanoaggregation and exhibits an 83-fold enhancement in conductivity and a 29-fold increase in toughness. Thanks to its excellent mechanical and electrical properties, this hydrogel can be used as a wearable supercapacitor and has potential applications as a high-performance soft electronic material in the fields of healthcare and robotics [103]. Zhai et al. also fabricated a strong and tough hydrogel with architected multiscale hierarchical structures through a freeze-casting-related strategy. The entangled crosslinked strong polyvinyl alcohol chains with chain-connecting ionic bonds, the hydrogen bond- and coordination bond-enhanced fibers with nanocrystalline domains, and the microscale anisotropic honeycomb-structured fiber walls and matrix constitute the multi-level hierarchical structures of the hydrogel and obviously enhance the mechanical properties of it [104].

Additionally, Lin et al. proposed a simple yet versatile method to construct hierarchically structured hydrogels through the flow-induced alignment of nanofibrils, as shown in Figure 3b–e. The obtained anisotropic hydrogels exhibit a highly aligned fibrous configuration and structural densification, leading to desired mechanical properties and damage-tolerant architectures. The strength can achieve 14 ± 1 MPa, and the toughness can achieve 154 ± 13 MJ m^−3^. These hydrogels also hold potential for water purification applications due to the ultra-fast and unidirectional water transport ability [105]. Furthermore, Zhu et al. developed a flexible, high-strength hydrogel with a fiberboard-and-mortar hierarchically ordered structure based on ultralong hydroxyapatite nanowires and polyacrylic acid, inspired by natural biological soft tissues. This hydrogel has a high water content, dense structures, and excellent mechanical properties similar to human cartilage, resulting in a huge potential for water purification, pollution treatment, biomedicine, nanofluidic devices, high-performance structural materials, and other applications [107]. As shown in Figure 3f, Velev et al. reported on a class of self-reinforced homocomposite hydrogels composed of sodium alginate interpenetrating networks of multiscale hierarchy. The storage modulus and Young’s modulus of these hydrogels are both enhanced by the colloidal network of hierarchically branched alginate soft dendritic colloids. They can even accomplish the efficient extrusion 3D printing of these hydrogels by balancing the ratio of the precursors [106]. Furthermore, Jeon et al. developed a method to prepare highly anisotropic hydrogels with programmable oriented polymer structures and excellent mechanical properties based on a novel welding technique for stretched cellulose hydrogel films. The anisotropic tough multilayer hydrogels obtained through this method can have different oriented hierarchies, as well as splendid mechanical properties (Young’s modulus of ~140 MPa, tensile strength of ~47 MPa). This work opens a new window for designing novel hydrogel materials utilized in engineering and biomedical applications [108].

### 2.3. Toughening Hydrogel with Network Topology

Constructing a uniformly dispersed network system or introducing mechanisms such as molecular entanglement [109] and molecular sliding into the network can significantly improve the mechanical properties of the hydrogels. For instance, Tetra-PEG hydrogel is formed by crosslinking four-armed polyethylene glycol modified with two different functional groups at the ends. It has been demonstrated to possess an extremely uniform network structure, possibly similar to the diamond structure, which significantly enhances its mechanical properties [33,110,111,112]. Wang et al. proposed chemically crosslinked microsphere composite hydrogels based on core-shell polymer microspheres formed by poly(butyl methacrylate-co-allylamine) (PBMA) with polyacrylamide (PAAm) chains chemically grafted onto their surfaces. The flexibility of the PBMA chains and the hydrogen bonds between the amide groups of the grafted polymer chains results in the reversible deformation of microspheres, providing an additional energy-dissipating mechanism for tough hydrogels. The good compatibility between the microspheres and the matrix polymer chains allows the hydrogel network structure to disperse uniformly, which is the main mechanism of toughening. In addition, hydrogen bonding between grafted polymer chains and reversible deformation of microspheres can also dissipate energy during the stretching process of this hydrogel [113]. Haraguchi et al. prepared Tetra-poly(ethylene glycol)-based nanocomposite hydrogels by in situ polymerization of two different four-armed macromonomers in the presence of clay in aqueous media. This hydrogel has high transparency, high elongation (900~1000%), and high tensile strength, which are approximately 2–4 times those of the corresponding original Tetra-PEG hydrogels [114]. Michelle et al. utilized electrospun gelatin nanofibres to produce aligned hydrogel networks, which are similar to the collagen network in natural tissues. This unique structure effectively resists crack expansion in the hydrogel and toughens it, making it suitable for various biomedical applications [115].

**Figure 4:**
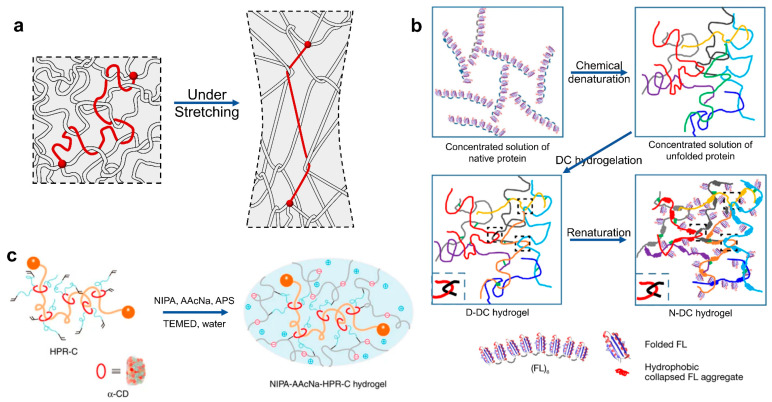
Schematic of high-toughness hydrogels toughening with network topology. (**a**) Schematic of tough hydrogels in which entanglements greatly outnumber crosslinks, adapted from Ref. [116]. (**b**) Schematics of the preparation of a denatured denatured-crosslinking hydrogel and a native denatured-crosslinking hydrogel. Reprinted with permission from Ref. [119]. 2023, Springer Nature. (**c**) A NIPA-AAcNa-HPR-C hydrogel containing slip ring structures. Reprinted with permission from Ref. [35]. 2014, Springer Nature.

Suo et al. studied the effects of polymer entanglements on fracture, fatigue, and friction. They synthesized polymer hydrogels in which entanglements greatly outnumbered crosslinks. Dense entanglements allow tension in the polymer chain to be transferred to many other chains, and the sparse crosslinked structures prevent the polymer chains from unraveling, as shown in Figure 4a. Thus, the hydrogels prepared by this method exhibit high toughness, strength, fatigue resistance, low hysteresis, low friction, and high wear resistance [116]. Gong et al. studied the effect of the molecular weight of the second network components on the entangled regions and proved that the entanglement between the second network component plays an important role in the toughening mechanism of double-network hydrogels [117]. In addition, Huo et al. prepared tough and recoverable nanostructured hydrogels through polymerization-induced self-assembly. The hydrophilic chains in its network structure are highly entangled and act as sliding links. The tension can be transferred to the micelle crosslinkers during the deformation process of the hydrogel, thereby dissipating energy through the reversible deformation and irreversible separation of the polymer chains and achieving toughening of the hydrogel [118]. Li et al. reported highly stiff and tough protein-based hydrogels constructed by introducing chain entanglements into the hydrogel network made of folded elastomeric proteins, as shown in Figure 4b. The exhibited mechanical properties are close to those of cartilage, and this hydrogel was proven to be useful in osteochondral defect repair [119].

Slide-ring hydrogels are composed of necklace-shaped macromolecule topological materials, which can be likened to a pulley that can slide randomly on a knotted chain. There are organized molecules at the end of the chain to prevent the pulley from falling off, so slide-ring hydrogels have strong tensile properties. Common cyclic molecules used include polyrotaxanes, catecholamine rings, and others, which are chemically modified to serve as sliding crosslinking points. The unique “sliding mechanism” of slide-ring hydrogels enables them to exhibit significantly different mechanical properties from traditional hydrogels, leading to a wide range of potential applications. For example, Yukikazu et al. used a polyrotaxane derivative composed of α-cyclodextrin and polyethylene glycol as a crosslinking point and polymerized it with a heat-sensitive N-isopropylacrylamide monomer, as shown in Figure 4c. They introduced ionic groups to promote the dispersion of slip-ring molecules in the system and constructed a NIPA-AAcNa-HPR-C hydrogel with slip-ring structures. It has strong tensile properties similar to soft rubber and can be used for rapid response thermosensitive and pH-sensitive materials [35]. Ichiro et al. demonstrated a novel procedure for synthesizing an aldehyde-containing polyrotaxane (PRβCD1), which is composed of β-cyclodextrin (βCD) monoaldehyde and polypropylene glycol. They also synthesized atelocollagen threads with enhanced mechanical properties by using PRβCD1 as a crosslinker (Col–PRβCD1) [120]. Moreover, Ito et al. proposed polyethylene glycol hydrogels crosslinked with moderate fractions of polymers that form sliding rings. These hydrogels exhibit high toughness and rapid recovery, owing to the rapid and reversible strain-induced crystallization facilitated by the parallel orientation of the chains when stretched [121].

### 2.4. Toughening Hydrogel with Force-Triggered Length Release

**Figure 5:**
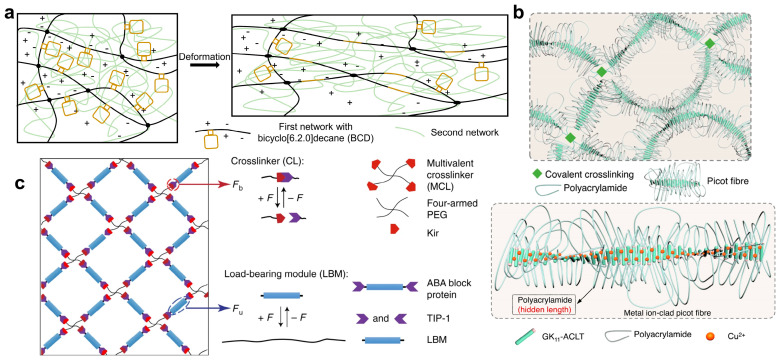
Schematic of high-toughness hydrogels toughening with force-triggered length release. (**a**) A double-network hydrogel with force-coupling structures, adapted from Ref. [125]. (**b**) A novel hydrogel comprised of hierarchical structures of picot fibers. Reprinted with permission from Ref. [126]. 2023, Springer Nature. (**c**) Schematic of a protein hydrogel with crosslinkers and folded proteins served as load-bearing modules. Reprinted with permission from Ref. [128]. 2018, Springer Nature.

Introducing force-triggered length release into the crosslinked network offers several benefits, including enhanced stress resistance, energy dissipation under large strains, and reversibility of deformation of the network [122,123,124]. This mechanism of toughening hydrogels is both innovative and effective. For instance, Craig et al. proposed a polymer network in which the constituent chains can be extended through force-coupling reactions when the chains reach their breaking point. This reactive chain extension only takes place when resistance to fracture is required, as shown in Figure 5a. As a result, the hydrogels exhibit significant toughening, achieving a 40% to 50% further extension through the unfolding of force-coupling structures under high strains, as well as double tearing energy [125]. Cao et al. presented novel types of hydrogels comprised of hierarchical structures of picot fibers, which were made of copper-bound self-assembling peptide strands with a zipped flexible hidden length, as shown in Figure 5b. The picots can efficiently dissipate energy and release the hidden length by rupturing strong inter-peptide interactions without affecting the network connectivity of the hydrogels. Consequently, the obtained hydrogels exhibit an extraordinary combination of high mechanical strength (fracture stress ~4.1 MPa), as well as ultrahigh toughness (fracture energy ~25.3 kJ m^−2^). Furthermore, the reformation of individual picot fibers occurs locally and independently, which ensures the rapid recovery of the hydrogels. This innovative design approach opens up new possibilities for advanced material applications [126]. Fei et al. constructed a super-tough polyacrylamide/iota-carrageenan double-network hydrogel with incorporated bacterial cellulose microclusters. This remarkable hydrogel can withstand tensile stress exceeding 200 kPa, possesses a high toughness of approximately 2000 kJ m^−3^, and achieves 27 times tensile strain. These exceptional mechanical properties are attributed to the intermolecular hydrogen bonds, the topological interlock, and the hidden length within the polyacrylamide regions [127].

Zheng et al. utilized 3D printing to create macroscopic super tough hydrogels featuring titin-like domains. When these hydrogels are stretched, the relatively thin and weak gel fibers break first, utilizing their hidden length to effectively delay the failure of the main structure. This mimics the toughening principle of titin and significantly enhances the tensile properties of the hydrogel [129]. Yang et al. proposed a hydrogel with high toughness, rapid self-healing abilities, dynamic adhesive properties, and strain hardening abilities by incorporating rigid tannic acid-coated cellulose nanocrystals into the polyvinyl alcohol-borax dynamic network. The flexible polyvinyl alcohol chains wrapped on the rigid cellulose nanocrystals can be stretched and release a significant hidden length when the hydrogel deforms. At the same time, there are dynamic hydrogen bonds between the tannic surface and the entangled polyvinyl alcohol chains. These two mechanisms synergistically toughen the hydrogel [130]. Babaahmadi et al. developed fully physically crosslinked double-network hydrogels, comprised of the robust agar biopolymer first network and the resilient polyvinyl alcohol second network. By employing cyclic freezing and thawing methods, they created a folded microcrystalline structure within the polyvinyl alcohol network, which unfolds as the hydrogel is stretched, thereby releasing the hidden length and toughening the hydrogel. This double-network hydrogel introducing a folded microcrystalline structure has multiple energy dissipation mechanisms, a high modulus of 2200 kPa, and a high toughness of 2111 kJ m^−3^ and can self-heal quickly [131]. Furthermore, introducing folded protein structures into hydrogel crosslinked networks is another approach to incorporate force-triggered length release. For example, Cao et al. demonstrated self-healable muscle-mimicking hydrogels that can significantly dissipate energy through protein unfolding, serving as load-bearing modules regulated by force, as shown in Figure 5c [128].

## 3. Applications of Tough Hydrogels

Hydrogels hold great promise in the biomedical field due to their high water content and excellent biocompatibility. However, traditional hydrogels often suffer from weak mechanical properties, limited functionalities, and a lack of self-healing capabilities, which restricts their applications. By designing complex functional structures within the hydrogel network, it is possible to imbue hydrogels with properties such as super stretch resistance, self-healing, high toughness, environmental sensitivity, conductivity, and so on. Then they can be widely used in tissue engineering, flexible electronic devices, and other biomedical fields, including biological scaffolds, drug delivery, load-bearing 3D printing biomaterials, wearable sensors, bioadhesive materials, and so on.

### 3.1. Tissue Engineering

**Figure 6:**
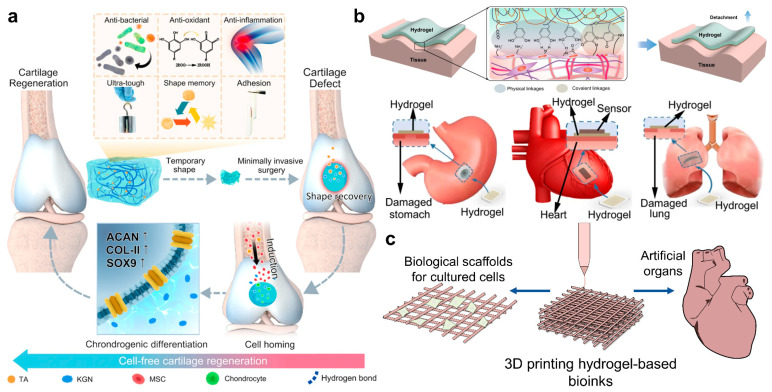
Applications of different high-toughness hydrogels in tissue engineering. (**a**) A multiple hydrogen-bond crosslinked hydrogel loaded with tannic acid and Kartogenin through polyaddition reaction, which can be used in cartilage regeneration in vivo. Reprinted with permission from Ref. [144]. 2023, Springer Nature. (**b**) Schematics of the adhesion mechanism and bioadhesion applications of a high-toughness hydrogel. Reprinted with permission from Ref. [148]. 2021, Springer Nature. (**c**) Schematics of 3D printing hydrogel bioink applied to cell culture scaffolds and artificial organs.

Tissue engineering is a multidisciplinary field focused on developing technologies that provide repair and replacement of damaged tissues and organs by studying factors such as cells, scaffold materials, and growth information [2]. Scaffold materials promote tissue regeneration by providing cells with a chemical environment and mechanical support. Tough hydrogels are expected to be used as a carrier for drug delivery or be used as a biological scaffold for cell culture in applications such as bone repair, cartilage repair, and cardiovascular tissue regeneration, especially after being special functionalized or 3D printed [57,132,133,134,135,136,137,138,139,140]. For example, Gong et al. introduced natural neutral polyelectrolytes into biopolymer-based tough double-network hydrogels by modifying them in polydimethylacrylamide crosslinked networks. The hydrogel’s mechanical strength and toughness are comparable to conventional double-network hydrogels. Meanwhile, it has excellent biocompatibility, and the cell experiments proved that this hydrogel can enhance the adhesion of human coronary artery endothelial cells (HCAECs) and promote cell proliferation, making it a promising candidate for tissue engineering applications, particularly in cardiovascular tissue repair [141]. Wear-resistant double-network hydrogels can be used as cell-free scaffolds for cartilage repair [142]. However, they face challenges in adhering tightly to the joint defect due to the strong water storage capacity. For example, Tan et al. developed a super-tough hydrogel that can be anchored to cartilage tissue via a dual-bonded approach by using chondroitin sulfate. The hydrogel implant stability with cartilage tissue is improved, and this hydrogel is favorable for chondrocyte attachment, offering the potential for rapid and effective repair of cartilage defects and long-term preservation of joint functionality [143]. In another study, Chen et al. proposed a multiple hydrogen-bond crosslinked hydrogel loaded with tannic acid and Kartogenin through a polyaddition reaction. As shown in Figure 6a, this hydrogel features ultra-durable mechanical properties, adequate adhesiveness with cartilage tissues, anti-inflammation, antibacterial capabilities, antioxidant, and fast shape memory properties, which endows the hydrogel with the potential for minimally invasive surgery. Importantly, the sequential release of tannic acid and Kartogenin can recruit the migration of bone marrow mesenchymal stem cells into the hydrogels and induce chondrocyte differentiation, thus leading to cartilage regeneration in vivo. This work may provide a promising solution for advancing cartilage regeneration [144].

In addition, hydrogels have potential applications as artificial bionic tissues for tissue replacement, tissue repair, and other fields if their mechanical properties are excellent enough, such as being very stiff and tough [119,145,146,147]. For instance, Li et al. designed a significantly stiffened protein-based hydrogel through chain entanglements, which exhibited high toughness, stiffness, fast recovery, and ultrahigh compressive strength. This hydrogel has been demonstrated for its application in cartilage regeneration [119]. Inspired by catechol chemistry discovered in mussel foot proteins, Cao et al. developed a high-toughness covalent hydrogel that can adhere to tissue surfaces through both physical and covalent interactions, as shown in Figure 6b. They have also successfully demonstrated that this hydrogel can be applied to stop bleeding in wounds in different tissues, including the stomach, heart, lungs, and so on [148]. David et al. developed a family of degradable tough adhesive hydrogels by incorporating covalently networked degradable crosslinkers and hydrolyzable ionically crosslinked main-chain polymers. These hydrogels have high fracture energies (>6 kJ m^−2^), excellent adhesion energies (>1000 J m^−2^), and good biocompatibility [149]. Kim et al. proposed a strong, stiff, and adhesive triple-network anisotropic hydrogel that can mimic a bone-adhering tendon. This anisotropic hydrogel is constructed based on the combination of imidazole-containing polyaspartamide and energy-dissipative alginate–polyacrylamide double-network, including ionic crosslinking interactions and covalent crosslinking interactions. It exhibits anisotropic, high tensile modulus and strength while maintaining high bone adhesion without chemical modification of the bone surface, benefiting from the secondary crosslinking after linearly stretched, similar to the nature of bone-adhering tendons [150]. In addition, Grunlan et al. demonstrated a double-network hydrogel composed of an anionic poly(2-acrylamido-2-methylpropanesulfonic acid) first network and a poly(N-isopropylacrylamide-co-acrylamide) second network. It exhibits similar compressive strength, modulus, and hydration with healthy cartilage, along with a lower coefficient of friction. Therefore, this hydrogel becomes an ideal candidate for synthetic cartilage grafts for chondral defect repair [151]. He et al. reported a soft somatosensitive actuating material composed of conductive photothermal responsive covalent hydrogel, synthesized through an ice-templated ultraviolet-cryo-polymerization technique. It has ultra-high conductivity, high stretchability, and quick response because of the dense conductive network and highly porous microstructure, which makes it a promising candidate for muscle-like materials [152].

Programmatic 3D printing of tough hydrogels with designing complex shapes, structures, and orientation-related properties may also make them be used as biological scaffolds for cultured cells, effectively reduce graft dislocation in medical treatment, better simulate the natural tissue environment, or be used as artificial tissues and organs in tissue engineering, as shown in Figure 6c [134,153,154,155,156,157,158,159,160,161]. These printable tough hydrogels were typically designed to demonstrate properties such as light-responsive fast curing for applications like stereolithography (SLA) 3D printing and shearing-thinning for processes such as extrusion printing. The strategies of sacrificial bonds were usually utilized to toughen hydrogels in 3D printing, including nanocomposite hydrogel, double-crosslinked hydrogel, and double-network hydrogel. For example, Liu et al. developed a nanocomposite hydrogel bioink consisting of a hydrogen-bonded monomer N-acryloyl glycinamide and nanoclay. This bioink can be 3D printed into high-strength composite scaffolds with excellent mechanical properties and swelling stability. The sustained release of intrinsic Mg^2+^ and Si^4+^ can effectively promote the repair of tibial bones in living mice [134]. In addition, Dai et al. proposed a stable, high strength, and 3D printable novel double crosslinked alginate hydrogel with enhanced mechanical properties and prolonged stability of up to 30 days in medium. This hydrogel holds promise for applications in cartilage defect repair and provides a possible approach for other tissue engineering and regeneration medicine [160]. Parker et al. designed a hydrogel ink containing prefabricated gelatin fibers, which can be used to print 3D organ-level scaffolds that recapitulate the intra- and intercellular organization of the heart [161]. These studies showcase the extensive possibilities of tough hydrogels in 3D printing technologies, opening up exciting prospects in the field of tissue engineering and regenerative medicine.

### 3.2. Flexible Electronics

**Figure 7:**
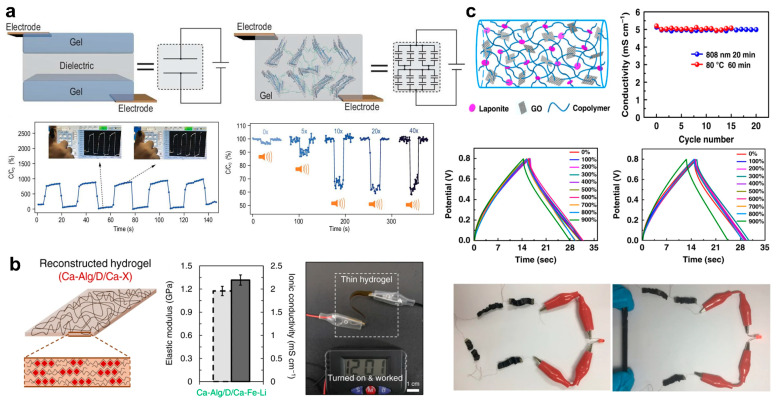
Applications of different high-toughness hydrogels in flexible electronics. (**a**) Single-layer composite hydrogels with bulk capacitive junctions, which can serve as mechanical sensors. Reprinted with permission from Ref. [171]. 2022, Oxford University Press. (**b**) Schematic, properties, and applications of a super strong, stiff, and conductive alginate hydrogel with densely interconnecting networks. Reprinted with permission from Ref. [172]. 2022, Springer Nature. (**c**) Schematic, properties, and applications of a hydrogel crosslinked by double linkers of Laponite (synthetic hectorite-type clay) and graphene oxide, which can serve as supercapacitors. Reprinted with permission from Ref. [173]. 2019, Springer Nature.

Stretchable tough hydrogels have the potential to be designed into various types of sensors when special structures that can respond to external environmental stimuli exist. These sensors can be used in wearable flexible electronic devices, including resistive sensors responding to pressure, capacitive sensors responding to strain, piezoelectric sensors, and others. Their high-stress threshold, good self-healing properties, and relative resistance to damage make them highly suitable for such applications [162,163,164,165,166,167,168,169]. For example, Alshareef et al. proposed an MXene-based hydrogel by incorporating a type of 2D early-transition metal carbides/carbonitrides Ti_3_C_2_T_x_ nanosheets (MXenes) with high conductivity and hydrophilic surface into polyvinyl alcohol hydrogel. This hydrogel has excellent tensile strain sensitivity, significant stretchability of more than 3400%, instantaneous self-healing ability, and excellent conformability. Therefore, it shows excellent sensing performance in sensing applications and can be used as a wearable electronic device to sense the movement of various parts of the body [170]. As shown in Figure 7a, Cao et al. designed single-layer composite hydrogels with bulk capacitive junctions as mechanical sensors. Dielectric peptide-coated graphene is engineered to serve as homogenously dispersed electric double layers in hydrogels. Thus, the overall capacitance can be significantly changed by any mechanical motions that alter the microscopic distributions of peptide-coated graphene in the hydrogels. Peptide self-assembly is used to render strong yet dynamic interfacial interactions between the hydrogel network and graphene, allowing the hydrogels to be stretched up to 77 times their original length and self-heal in a few minutes [171].

Hydrogels with integrated conductive structures also hold great potential as conductive materials for use in brain-computer interfaces due to their good biocompatibility [174,175,176,177]. For example, Zhao et al. proposed a bi-continuous conducting polymer hydrogel, which simultaneously exhibits high electrical conductivity, stretchability, and fracture toughness. This hydrogel can also be 3D printed and used as a bioelectronic interface material to record and stimulate the long-term electrophysiological in rat models [178]. Another example is the protein-based bioprotonic hydrogel designed by Mao et al., which boasts reliable water retention capacity, antifreeze ability, stretchability, transparency, and biodegradeability. This hydrogel can serve as an artificial bioprotonic skin for the long-term collection of high-quality human electrophysiological signals and self-powered sensing, which may even promote the development of novel human–machine merging interfaces [167]. In addition, Kim et al. demonstrated super strong, stiff, and conductive alginate hydrogels with densely interconnecting networks. They achieved this by anisotropic densification of Ca-Alg pre-gel and a subsequent ionic crosslinking with rehydration, as shown in Figure 7b. These hydrogels demonstrate exceptional tensile strengths (8–57 MPa) and elastic moduli (94–1290 MPa). Additionally, they exhibit strong interfacial adhesion, making them suitable as stable gel electrolyte membranes for use in aqueous supercapacitors [172].

Hydrogel flexible electronics also show promising applications in flexible energy storage devices [179,180,181,182,183,184]. For example, Guo et al. integrated a carbon nanotube conductive network and an electrolyte salt into a flexible double-network hydrogel to create a hydrogel supercapacitor. This supercapacitor can deliver high areal capacitance and maintain stable energy output under various deformations [185]. In addition, Xie et al. constructed a surface-microstructured tough hydrogel electrolyte composed of agar/polyacrylamide/LiCl (AG/PAAm/LiCl). By attaching activated carbon electrodes, they successfully prepared stretchable supercapacitors capable of maintaining good electrochemical behavior and capacitance under large mechanical strains [186]. In addition, Chen et al. proposed a hydrogel of a copolymer crosslinked by double linkers of Laponite (synthetic hectorite-type clay) and graphene oxide, as shown in Figure 7c. This hydrogel exhibits high mechanical stretchability, excellent ionic conductivity, and superior healable performance [173]. The supercapacitors fabricated using this hydrogel as a gel electrolyte demonstrate repeated healable performance under treatments of both infrared light irradiation and heating, as well as stable electrical properties under stretching. It is worth noting that this supercapacitor has a stretchability of 900% with slightly reduced performance, even if it has defects.

## 4. Outlook

High strength, toughness, and rapid recovery are inherent properties of many natural materials, such as biological tissues [187,188,189,190]. These properties have inspired the exploration of synthetic materials, particularly hydrogels, which have a wide range of potential applications, from biomedical devices to flexible electronic devices [191,192,193,194,195,196]. However, there are still many challenges in incorporating these properties into hydrogels due to the unique network structure and unique mechanical response of hydrogels. For example, in terms of preparation methods, more in situ gelation methods need to be developed to meet the need for rapid solidification of hydrogels in 3D printing. In terms of application scenarios, integrating anti-dehydration mechanisms such as grafted surface coatings would allow hydrogels to be stably stored long-term in various conditions and expand their application scope. Experimental, theoretical, and computational methods are indispensable tools in this pursuit. Simulation and modeling can provide insights into and predict the microstructural evolution and mechanical behavior of materials [197,198]. Finite element analysis and molecular dynamics simulations are particularly useful for understanding the interactions between various network characteristics and mechanical properties [171,199,200,201,202,203]. With the development of artificial intelligence (AI), machine learning can be used to train models on large experimental and simulated datasets to predict the composition and manufacturing conditions of optimal hydrogels [204,205,206]. After optimizing and improving the mechanical performance of hydrogels using AI, the manufacture of novel hydrogels can be achieved through proposed structural designs, significantly speeding up the development process.

The potential applications of these advanced tough hydrogels are vast and diverse. In the field of medicine, they can revolutionize skin repair techniques, serve as efficient artificial muscles for prosthetic limbs, enable precise drug delivery systems for targeted therapies, and provide scaffolding for tissue engineering, promoting the regeneration of damaged tissues. In the realm of soft robotics, their tunable mechanical properties make them versatile components that make them capable of functioning as actuators to drive movements, as sensors to detect environmental changes, or even as materials for human-bio interface applications, enhancing the interaction between humans and machines. Furthermore, their inherent self-healing abilities hold promise for creating durable and reusable materials across various industries, contributing to sustainable and cost-effective solutions. In addition, the fusion of tough hydrogel materials with synthetic biology has emerged as a leading force in the realms of biodegradable and reproducible functional materials, which are also so-called living hydrogels [207,208,209,210,211]. For example, Zhao et al. present a design for stretchable, robust, and biocompatible hydrogel–elastomer hybrids hosting various genetically engineered bacterial cells, combing the stretchability and robustness of tough hydrogels and biological functions of living bacterial cells [210].

In summary, by drawing inspiration from nature and leveraging experimentation, simulation, and artificial intelligence, hydrogels with high strength, toughness, and rapid recovery can be rationally designed and achieved. Future trends in hydrogel preparation will focus on creating tough, multifunctional, intelligent, and living materials, opening up new avenues in science and technology.

## Data Availability

All data are available in the main text.
